# Bilateral vocal fold immobility: a 13 year review of etiologies, management and the utility of the empey index

**DOI:** 10.1186/s40463-015-0080-8

**Published:** 2015-06-26

**Authors:** Maria K. Brake, Jennifer Anderson

**Affiliations:** Department of Otolaryngology – Head and Neck Surgery, University of Toronto, Ontario, Canada; St. Michael’s Hospital, Department of Otolaryngology – Head and Neck Surgery, University of Toronto, 30 Bond St. 8C-129, ON M5B 1 W8 Toronto, Canada

**Keywords:** Bilateral vocal fold immobility, Bilateral vocal cord immobility, Glottis stenosis, Cordotomy, Arytenoidectomy, Empey, Expiratory disproportion index

## Abstract

**Background:**

Bilateral vocal fold immobility (BVFI) is a rare diagnosis causing dyspnea, dysphonia and dysphagia. Management depends on respiratory performance, airway patency, vocal ability, and quality-of-life priorities. The authors review the presentation, management and outcome in patients diagnosed with BVFI. The utility and efficacy of the Empey index (EI) and the Expiratory Disproportion Index (EDI) are evaluated as an objective monitoring tools for BVFI patients.

**Methods:**

A 13-year retrospective review was performed of BVFI patients at St. Michael’s Hospital, University of Toronto, a tertiary referral centre for laryngology.

**Results:**

Forty-eight patients were included; 46 presented with airway obstruction symptoms. Tracheotomy was required for airway management in 40 % of patients throughout the course of their treatment, which was reduced to 19 % at the end of the study period. Twenty-one patients underwent endoscopic arytenoidectomy/cordotomy. Non-operative management included continuous positive airway pressure devices. Pulmonary function testing was carried out in 29 patients. Only a portion of the BVFI patients met the defined upper airway obstruction criteria (45 % EI and 52 % EDI). Seven patients had complete pre- and post-operative PFTs for comparison and all seven had ratios that significantly improved post-operatively which correlated clinically.

**Conclusion:**

The EI and EDI have limited use in evaluating patients with who have variable upper airway obstruction, but may be helpful in monitoring within subject airway function changes.

## Background

Bilateral vocal fold immobility (BVFI) is a rare diagnosis that can be due to paralysis or fixation of the vocal folds, and frequently associated with significant morbidity and disability. Depending on the underlying etiology, vocal fold position and compensatory behaviour, varying degrees of dyspnea, dysphonia and dysphagia occur [1–3].

Determining the need for surgery, as well as assessing outcomes can be difficult due to the variability in etiology, symptoms and limited BVFI patient population. Investigations available to assess the airway function include imaging, physical examination with endoscopy, pulmonary function testing (PFT), sleep studies and validated quality of life questionnaires. Parameter ratios of individual PFT values have been proposed as a potential objective measure of upper airway function. One such measure is the Empey Index (EI), which was described in 1972 as a marker of upper airway obstruction by Duncan Empey [4–6]. The index is the ratio of forced expiratory volume in 1 s (FEV_1_) in milliliters to the peak expiratory flow rate (PEFR) in litres per minute. The respiratory physiology is described elsewhere [4–6] but can be summarized as follows:

*The ratio of FEV*_*1*_*is predominantly determined by the properties of the small intrathoracic airways and less affected by upper airway stenosis than PEFR. In the setting of upper airway stenosis, the ratio increases.*

In normal subjects and in patients with lung diseases (asthma, chronic bronchitis and others) the EI ratio was found to be less than 10 (ml/l/min). In comparison, Empey discovered that patients with known upper airway obstruction, including those with bilateral vocal fold fixation, all had values greater than 10 and a mean of 14 [4].

When Nouraei *et al.* re-evaluated this ratio in 2013, they proposed a modified ratio calculation of FEV_1_/PEFR × 100, termed the expiratory disproportion index (EDI), using the SI units of FEV_1_ (L) and PEFR (L/s) [6]. In studying the utility of the EDI, they found that benign upper airway stenosis subjects had a mean of 76 ± 17 s. Their study also found that the degree of stenosis correlated with a higher EDI value. For practicality, multiplying EI ratio by six will allow conversion to EDI units.

It is not clear whether these ratios can be useful in the BVFI population, which includes a large proportion of bilateral vocal fold paralysis patients. In this report, the authors review the presentation, management and outcome in patients diagnosed with BVFI at a laryngology clinic in a tertiary referral academic centre. Within this group, the utility and efficacy of the EI and EDI are evaluated as an objective monitoring tool for BVFI patients.

## Material and methods

A retrospective cohort study was undertaken at an academic, tertiary-care laryngology clinic in Toronto, Canada. All patients diagnosed with BVFI between January 1, 2001 and Dec 31, 2013 were included. Collected data included demographics (age, gender), etiology of BVFI, past medical history, pulmonary function testing (flow volume loop, FEV1 and PEFR), relevant surgeries, history of tracheotomy, decannulation, and post-operative complications. The hospital electronic medical record system was used to review transcriptions, clinic notes, pathology reports, operative notes and pulmonary function results to collect the data.

The data was analyzed using GraphPad Prism® version 6.02. Descriptive statistics were calculated for demographics, EI, and EDIs ratios. The Wilcoxon Matched Pairs Signed Rank Test was used to compare the paired pre- and post- operative EIs and EDIs. Unpaired parametric t-tests were used to compare the EI/EDI ratios between mean ages patients that underwent airway surgery, specifically a cordotomy/artenoidectomy, versus the group that did not. Chi-squared testing was used to determine if the rates of cordotomies, tracheostomy or decannulation varied between number of cormorbities. In all cases *p* < 0.05 was considered statistically significant. Ethics approval was obtained from the St. Michael’s Hospital Research Ethics in Toronto, Canada.

## Results

A total of 48 patients with bilateral vocal fold immobility were identified from the institutional laryngology database. The mean age of presentation was 52 years, with ages ranging from 15 – 83 years. Thirty-five (73 %) of the patients were female. Thirty-three of the patients (69 %) were diagnosed with bilateral vocal fold paralysis, twelve (25 %) with joint fixation, and one was documented as a combination. Two patients were unable to be categorized given the information available. Seven patients (17 %) were documented as smokers. Demographics are outlined Table 1.

**Table 1:** Patient demographics bilateral vocal fold immobility (*n* = 48)

Demographics (*n* = 48)	n (%)
Gender	
Male	13 (27 %)
Female	35 (73 %)
Documented Smokers	7 (17 %)
Diagnosis	
Bilateral Vocal Fold Paralysis	33 (68 %)
Joint Fixation	12 (25 %)
Combination	1 (2 %)
Unknown	2 (4 %)
Presentation	
Airway Obstruction	36 (75 %)
Dysphonia	6 (13 %)
Dysphagia/Aspiration	2 (4 %)
Other	2 (4 %)
Unknown	2 (4 %)

### Presentation

The primary complaint for the majority of patients (75 %) presenting to the clinic was that of dyspnea such as stridor, exercise intolerance, and airway obstruction when supine. Vocal complaints were the presenting complaint in 6 patients (12.5 %), followed by two with dysphagia or aspiration (4 %). Two patients (4 %) were seen on the recommendation of the referring doctor but reported to be without symptomatic complaints otherwise.

### Etiology

**Fig. 1:**
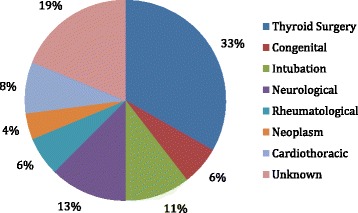
Etiologies of BVFI in the study population

The etiology of BVFI in the study population, in decreasing order of incidence, included thyroid disease or associated surgery, intubation-related injury, congenital, central neurological disorders, autoimmune disease, and surgery or cancer of cardiothoracic origin (Fig. 1).

Of the sixteen patients who suffered from bilateral vocal fold paralysis secondary to thyroid surgery, it was noted that 44 % (7) had their surgery outside of Canada and the pathology could not be determined.

A head and neck cancer diagnosis was present in eleven (23 %) patients. Four of these patients (36 %) had BVFI secondary to direct malignant invasion of the nerve or larynx. The other seven (64 %) developed BVFI from cancer treatment interventions: three with post-radiation polyneuropathy and four from injury or intentional transection during surgery for their disease.

### Tracheotomy

Nineteen BVFI patients (40 %) required a tracheotomy for airway management at some point in their management. Nine patients with tracheotomy opted to undergo further surgical treatment with the intent to decannulate. To date, eight of these have been decannulated at the time of manuscript preparation.

**Fig. 2:**
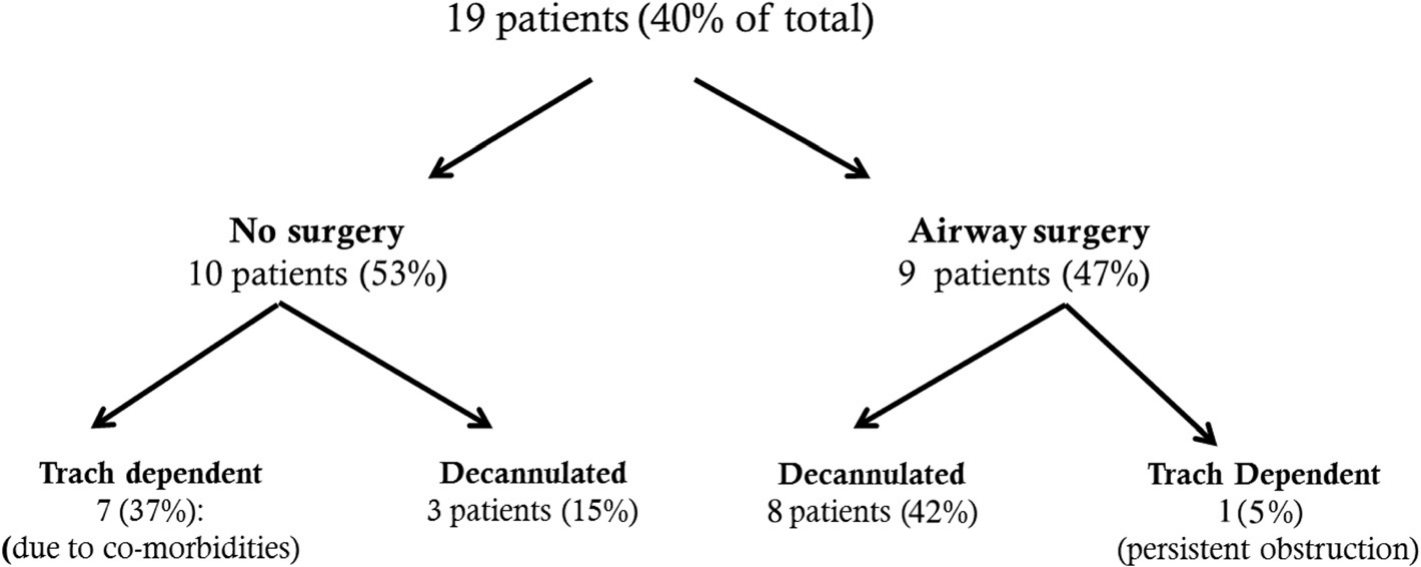
Patients requiring a tracheostomy

Three patients were decannulated without surgical intervention. Overall, 17 % (8) of the 48 BVFI patients continued to require a tracheotomy for airway control (see Fig. 2 for full details). There was no statistical significance between the tracheostomy-dependent groups, the groups who were decannulated or the groups that did not require tracheostomies in terms of ages or comorbidities.

### Surgical intervention

Of the 46 patients who complained of airway obstruction, twenty one patients (44 %) underwent a unilateral cordotomy and arytenoidectomy in order to improve the upper airway. The remaining 25 patients either had mild symptoms or medical comorbidities that prohibited a cordotomy/arytenoidectomy.

Revisions were required in seven (33 %), including five patients with persistent airway symptoms and two due to granuloma formation. Of the patients requiring revisions, four (44 %) of the seven had BVFI secondary to fixation: two cases of post-glottic stenosis, one case of a Teflon granuloma, and a fourth case secondary to SLE-related joint fixation.

**Fig. 3:**
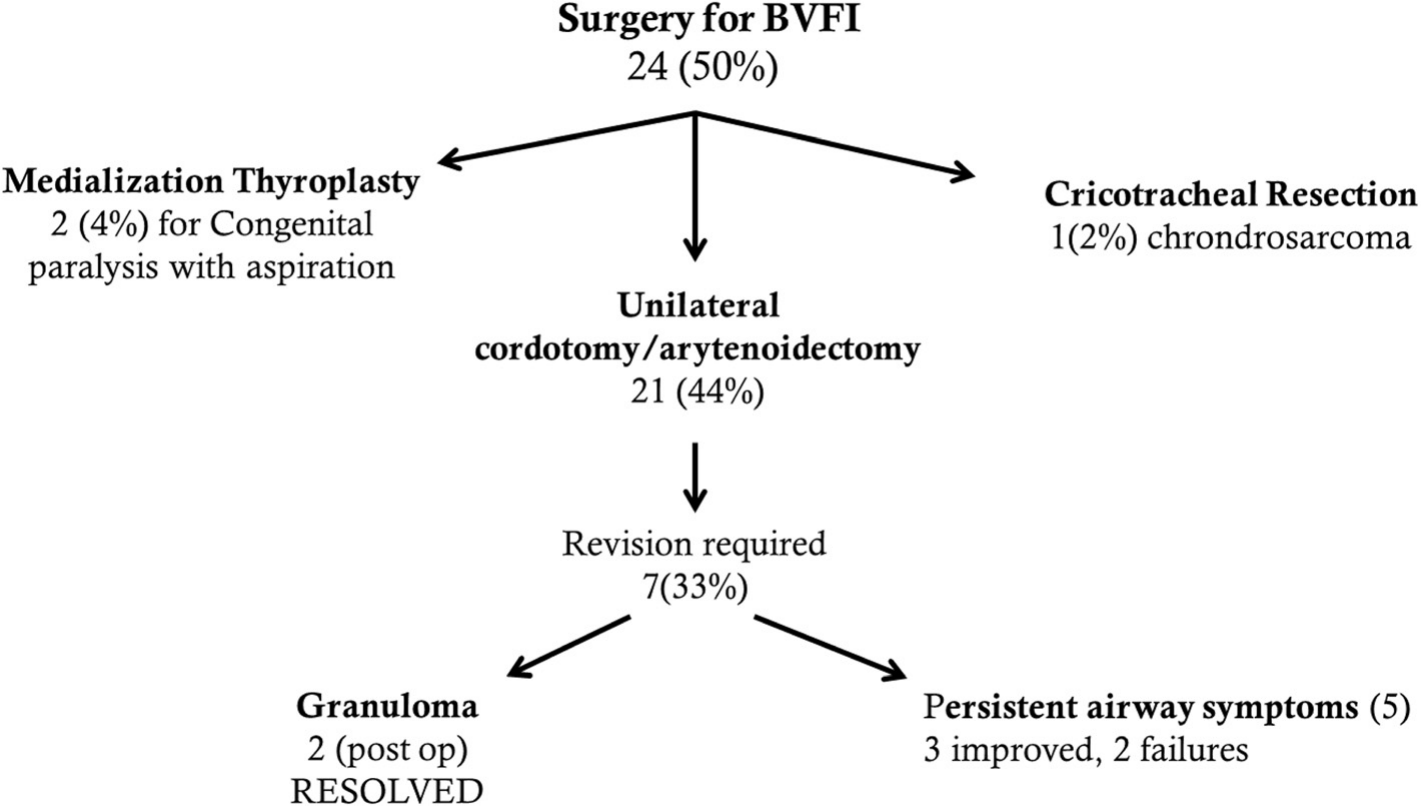
Surgical interventions

Two patients required multiple revisions (see the flow chart in Fig. 3 for full details). In one instance the patient had notable weight gain and recurrence of Reinke’s edema in the setting of paralysis attributed to Charcot-Marie-Tooth disease. This individual required a contralateral cordotomy/arytenoidectomy, followed by an external approach vocal cord lateralization via laryngofissure. After the final procedure, the patient was able to achieve decannulation. The second patient had the aforementioned Teflon granuloma and associated sequelae.

There was no significance between cordotomy and non-cordtomy groups in terms of ages or comorbidities; there was also no significance for these same variables when comparing the revision cordotomy groups to the single-surgery patients.

Two patients underwent medialization thyroplasty for lateralized vocal folds in order to improve vocal function and reduce aspiration. In both of these cases, the etiology of their BVFI was congenital resulting in widely patent glottis and poor voice.

### Empey ratio

A total of 81 PFTs had been completed on 60 % (29) of the patient population. The surgical group (*n* = 21) had 49 PFTs done in total, with this group referring specifically to patients having undergone a cordotomy/arytenoidectomy and/or revision. In the non-surgical group (*n* = 25), 32 PFT studies were done as part of their evaluation. Only twelve (41 %) of the 29 patients who underwent full PFT studies met the defined EI criteria for upper airway obstruction. Fifteen patients (52 %) had EDI values which fell within the mean range of 76 ± 17 as described by Nouraei *et al.* [4].

The EI and EDI were separated into surgical and non-surgical groups for comparision. Within the surgery group, comparision was also completed between subgroups: last pre-operative value, first post-operative value, all pre-operative values and all post-operative values. It should be noted that some patients only had pre-operative values while some others only had post-operative values. There was no significance found between the mean EI and EDI ratios in the non-surgical and surgical groups, nor between any of the subcategories.

Seven patients had complete PFT data allowing for comparison from pre to post cordotomy/arytenoidectomy. The Wilcoxon Matched-Pairs Signed Rank Test (WSRT) was completed on the Empey ratios and EDI for this subgroup which showed a significant increase post-surgery (*p* = 0.0156), which also correlated with clinical airway improvement. One patient was initially found to have a post-operative granuloma, worsening dyspnea and an elevated EI (10.53) and EDI (63.17). A PFT was repeated following revision surgery and the final value was used as the post-operative value for comparision.

Nineteen patients did not have records of PFTs in their charts; two had medical reasons for not pursuing further surgical treatment (both had long term tracheotomy) and three others had mild symptoms that did not warrant intervention. Other patients could not complete the PFT tasks due to airway limitations (with a corked tracheotomy tube). It is unclear how many PFTs were performed at outside institutions and were not added to our institutional electronic medical record.

### Other management

The majority of patients (56 %) underwent laryngeal computer tomography. Ten patients of 46 (21 %) were recommended to use home continuous positive airway pressure (CPAP) devices secondary to the diagnosis of obstructive sleep apnea on the basis of abnormal sleep studies attributable to their upper airway pathology. Of these patients, five patients were post-operative from a cordotomy/arytenoidectomy and the remaining five patients did not undergo any surgical airway intervention.

## Discussion

Airway obstruction is the most disabling symptom in patients with BVFI. In both glottic stenosis and bilateral vocal fold paralysis, the primary goal is to provide a stable increase in glottic airway with minimal compromise in voice quality. Airway management depends on a variety of factors, including their degree of upper airway obstruction, underlying respiratory function, voice demands and quality of life priorities. Long-term tracheostomy, which was the only treatment available until 1922 [7–9], may be an appropriate option in some patients for either quality of life preference (voice quality) or for medical reasons. Other patients may choose to undergo surgical procedures in order to improve glottic airway patency, potentially at the risk of increased dysphonia and aspiration, in order to have the tracheotomy tube removed.

Chevalier Jackson was the first to introduce surgical treatment in BVFI via an open approach cordectomy and ventriculocordectomy [10]. Poor voice and swallowing outcome prompted modifications: submusocal resection of the true vocal cord [11], extralaryngeal arytenoidectomy with arytenopexy to the ipsilateral strap muscles [12], ^,^arytenoidectomy via a thyroid alar window [13], and arytenoid abduction with lateral suture by Woodman [14]. Techniques were further improved with the introduction of the transoral arytenoidectomy in 1948 by Thornell [15]^,^ and the application of endoscopic carbon dioxide laser [16–18].

Dennis and Kashima popularized the use of endoscopic posterior glottic cordotomy in 1989, which remains the most common surgical option, either as a unilateral [19] or bilateral intervention [20]. Other reports have been published with variations on these procedures including suture techniques [21–23], mucosal approaches [24] and equipment [10].

In our cohort, bilateral vocal fold immobility was most frequently caused by bilateral recurrent nerve paralysis after thyroid surgery, (Fig. 1). A twenty –year review of the etiology of bilateral vocal fold immobility was completed by at Cleveland Clinic in [25]. The percentages of BVFI attributed to thyroid surgery, intubation, cardiothoracic malignancy, neurological disease and radiation were within 3 % or less of the rates quoted in the 20-year review; our cohort also had a higher rates of idiopathic/unknown causes (19 % vs 11.4 %) as well as rheumatologic disease (6 % vs 2.7 %).

The majority of patients (75 %) presented with complaints of dyspnea secondary to paramedian positioned vocal folds and inadequate airway. Rarely, patients can present with bilaterally lateralized vocal folds; in our case two patients presented in this fashion and underwent Type I thyroplasty. Both of these patients were found to have congenital etiologies, suggesting a high vagal or central pathology other than the recurrent laryngeal nerve dysfunction.

Many patients in our series did not require surgery due to mild symptoms, and were monitored clinically via regular clinical examinations which included laryngoscopy and serial PFTs.

The decision to treat is based on the severity of symptoms, functionality and patient priorities. For some, optimal vocal quality is worth living with a tracheostomy, but for most, decannulation of their tracheotomy, or improvement of their airway obstruction symptoms is the primary goal of treatment. Of the 21 patients who underwent a cordotomy, 2 (10 %) failed to improve and continued to required tracheotomy, in keeping with the literature in this population [2, 9]. No major complications occurred in the surgical treatment group.

CPAP has been shown to effectively manage sleep apnea without surgical intervention in patients with bilateral vocal cord paralysis and tracheal stenosis [26]. Our patients who are borderline in terms of symptoms or those who are unwilling or unfit to undergo a permanent airway modifying surgery may benefit from nocturnal CPAP. Sleep studies are often performed on these patients, as well as those undergoing corking trials, prior to decannulation [27].

Pulmonary function testing is currently proposed as the one of the most useful objective evaluation of the respiratory system, although application to upper airway obstruction has been problematic both as a diagnostic and quantitative measure. The ratio of FEV1 and PEFR expressed either as EI or as EDI, has been proposed as a useful tool in identifying upper airway obstruction [4, 6].

In our cohort, surprisingly, only half of the patients (41 % of EI/52 % of EDI) met the published criteria for upper airway obstruction, despite the diagnosis of BVFI and 96 % having presented with variable degrees of airway symptoms. The proposal by Nourai [4] to utilize EDI as a diagnostic tool for upper airway obstruction was based on PFT results in laryngotracheal stenosis patients (*n* = 184). However, the pathophysiology of glottis obstruction is unique in bilateral vocal fold paralysis wherein the inspiratory flow is more adversely affected than the expiratory flow based on the Bernoulli effect [28]. This rationale may explain why our BVFI cohort, consisting of predominantly bilateral vocal fold paralysis, were found to have a much lower mean ratio of EDI (52) than was reported by Nourai in fixed obstruction patients (76). Since bilateral vocal fold paralysis is predominantly an inspiratory obstruction, It may be more appropriate to quantified bilateral vocal fold paralysis using a ratio containing an inspiratory parameter, such as peak inspiratory flow rate (PIFR).

Regardless, the subgroup of seven patients who underwent a cordotomy/arytenoidectomy and who also had complete pre- and post- PFT data, did show a statistically significant increase in EDI/EI postoperatively, which also correlated with clinical airway improvement. Although it is difficult to draw a conclusion regarding the utility of this ration based the small sample size, these findings do suggest these proposed ratios still may have some utility in objectively monitoring patients’ airway status.

In bilateral vocal fold paralysis, emerging research has shown that in a highly selected patients, re-innervation of the recurrent laryngeal nerve using a phrenic nerve rootlet maybe be effective in providing active abduction [29]. There is also some evidence that transecting the RLN branch between the PCA and interarytenoid may prevent aberrant reinnervation and help avoid synkinesis; other have taken the approach of selectively reinnervating the adductors and the PCA muscles separately, with goals of isolated abduction during respiration while maintaining good adduction for voice production [28]. Reinnervation techniques of the RLN are still primarily in the research stage, but there are positive preliminary outcomes suggesting promise for bilateral vocal cord paralysis patients in the future, particularly in younger BVFI patients.

## Conclusion

Bilateral vocal fold immobility is a challenging condition to manage due to the significant morbidity associated with the condition. Adequate airway management using endoscopic cordotomies/arytenoidectomy can achieve symptom improvement and decannulation for the majority of patients. CPAP ventilation can be a useful adjunct for those patients who continue to experience upper airway obstruction while sleeping.

Pulmonary function testing has much potential for the utility in the monitoring of these patients but ideal parameters in the setting of upper airway obstruction is still unclear. In our cohort of BVFI patients, EI/EDI ratios were not overly reliable in identifying bilateral vocal cord immobility, unlike in previously published reports. This different could be due to anatomic differences of BVFI versus the previous studies, which were based on subglottic stenosis – a fixed obstruction. Additional prospective data collection on this patient population, including evaluation of parameter ratios that include PFT inspiratory flow values may help us to further understand the utility of pulmonary function testing in objectively monitoring patients with obstructive symptoms secondary to bilateral vocal cord immobility.
